# A functional requirement for sex-determination M/m locus region *lncRNA* genes in* Aedes aegypti* female larvae

**DOI:** 10.1038/s41598-021-90194-7

**Published:** 2021-05-20

**Authors:** Keshava Mysore, Limb K. Hapairai, Ping Li, Joseph B. Roethele, Longhua Sun, Jessica Igiede, Joi K. Misenti, Molly Duman-Scheel

**Affiliations:** 1 Department of Medical and Molecular Genetics, Indiana University School of Medicine, Raclin-Carmichael Hall, 1234 Notre Dame Ave., South Bend, IN 46617 USA; 2 University of Notre Dame Eck Institute for Global Health, Notre Dame, IN USA

**Keywords:** Genetic techniques, Genomic analysis, Biological techniques, RNAi, Infectious diseases, Viral infection, Functional genomics, Biotechnology, Genome evolution, Developmental biology, Genetics, Development, Evolutionary biology, Functional genomics, Microbial genetics, RNAi

## Abstract

Although many putative *long non-coding RNA (lncRNA)* genes have been identified in insect genomes, few of these genes have been functionally validated. A screen for female-specific larvicides that facilitate *Aedes aegypti* male sex separation uncovered multiple interfering RNAs with target sites in *lncRNA* genes located in the M/m locus region, including loci within or tightly linked to the sex determination locus. Larval consumption of a *Saccharomyces cerevisiae* (yeast) strain engineered to express interfering RNA corresponding to lncRNA transcripts resulted in significant female death, yet had no impact on male survival or fitness. Incorporation of the yeast larvicides into mass culturing protocols facilitated scaled production and separation of fit adult males, indicating that yeast larvicides could benefit mosquito population control strategies that rely on mass releases of male mosquitoes. These studies functionally verified a female-specific developmental requirement for M/m locus region *lncRNA* genes, suggesting that sexually antagonistic *lncRNA* genes found within this highly repetitive pericentromeric DNA sequence may be contributing to the evolution of *A. aegypti* sex chromosomes.

## Introduction

Female mosquitoes differ from males in morphological, physiological, and behavioral traits, such as blood feeding behavior, that promote the spread of disease-causing pathogens. Although genes that regulate female-specific traits may represent novel targets for vector control, a majority of these genes have not yet been functionally characterized in mosquitoes, including *Aedes aegypti,* the primary vector for arboviruses that cause Zika, chikungunya, yellow fever, and dengue^1^. Moreover, population-based strategies for mosquito control, including the sterile insect technique and the incompatible insect technique, are often dependent on the mass release of adult male mosquitoes^2^. The identification of genes with female-specific functions could also permit the elucidation of genetically-based effective, affordable, and scalable mosquito sex-sorting technology that can be readily deployed worldwide, which would facilitate global implementation of emerging population-based mosquito control strategies^2^.

*A. aegypti* sex determination is regulated by a non-recombining Y-chromosome-like male determining M locus (Supplementary Fig. S1), which has a pericentromeric location on chromosome one^3,4^ and contains the male-determining factor *Nix*^5^. Males, which possess one copy of the chromosome bearing the M locus and one which lacks it, have an M/m genotype, while females, which lack the male determining locus, are m/m^6^. Although the *A. aegypti* M and m sex chromosomes are homomorphic, the sex-differentiated region extends to a ~ 100 Mb region that surrounds the ~ 1.5 Mb M locus^7^. Rare recombination events in the M locus region, in which recombination is typically suppressed, result in sex ratio distortion^8,9^. These distortions suggest that clusters of loci which cause sex-specific effects reside within the sex-determining region and are gained or lost through crossover events, causing sex-specific lethality^8,9^. The factors, which may include genes that are vital for development or which are sexually antagonistic, may be shaping the stable boundaries of non-recombining sex chromosomes during *A. aegypti* sex chromosome evolution^9^, but the identities of these loci are unknown.

Although characterization and sequencing of the M/m locus had been thwarted by the repetitive nature of DNA located in this region, recent innovations in sequencing technology generated an improved and re-annotated genome assembly that facilitated better estimation of the M/m locus^7^. The improved sequence revealed the presence of many putative *long non-coding RNA (lncRNA)* genes in the M/m locus sex-determining region^7,10^. lncRNAs are a class of non-coding transcripts that are > 200 nucleotides in length which are not translated into proteins^11^. lncRNAs regulate a wide array of cellular activities, such as the recruitment of chromatin modifiers and transcription factors, the regulation of chromosome looping, microRNA sequestration, and translational control^12^. Although thousands of putative lncRNAs have been annotated in insect genomes^13^, including *A. aegypti*^10,14–16^, very few have been functionally validated as lncRNAs. Two of the most well-characterized insect lncRNAs, *Drosophila melanogaster roX1* and *roX2*, function as components of the Male Specific Lethal (MSL) complex, which regulates dosage compensation by upregulating X-linked gene expression in male fruit flies^17,18^. Mammals regulate dosage compensation through random inactivation of one X chromosome, a process that is regulated by *X inactive specific transcript (Xist),* an lncRNA which promotes chromatin silencing^19,20^. Given the critical sex-specific roles of these lncRNAs, it was hypothesized that *A. aegypti* M/m locus region *lncRNA* loci encode functional transcripts that have evolved sex-specific functions.

In this investigation, a small interfering RNA (siRNA) screen for female-specific larval lethal genes uncovered multiple *lncRNA* genes located at or tightly linked to the M/m locus, a chromosomal location herein referred to as the M/m locus region (Supplementary Fig. S1). Silencing several of these transcripts with yeast interfering RNA technology revealed a female-specific requirement for M/m locus region lncRNAs in larvae that could be exploited for the development of scalable male sex separation strategies. These findings suggest that *lncRNA* genes in the M/m locus region may be contributing to sex chromosome evolution in *A. aegypti.*

## Results and discussion

### A screen identifies siRNAs that induce female-specific larval mortality

Recent high-throughput screens in which first instar (L1) larvae were briefly soaked in siRNAs led to the discovery of hundreds of protein-coding larval lethal genes and a new class of RNAi-based mosquito insecticides^21–23^. Given that the M/m locus is believed to be tightly linked to developmental genes that confer sex-specific lethal effects in *A. aegypti*^8,9^, *lncRNA* loci located both within, as well as flanking the M/m locus, were evaluated in a female larval lethal soaking screen that employed a similar strategy. These studies permitted functional assessment of the hypothesis that silencing *A. aegypti* M/m locus region *lncRNA* genes during larval development would induce female-specific lethality. A total of 50 siRNAs corresponding to *lncRNA* genes in and flanking the M/m locus, which is referred to herein as the M/m locus region (Supplementary Fig. S1), were screened (Supplementary Tables S1, S2, S3, S4).

**Figure 1:**
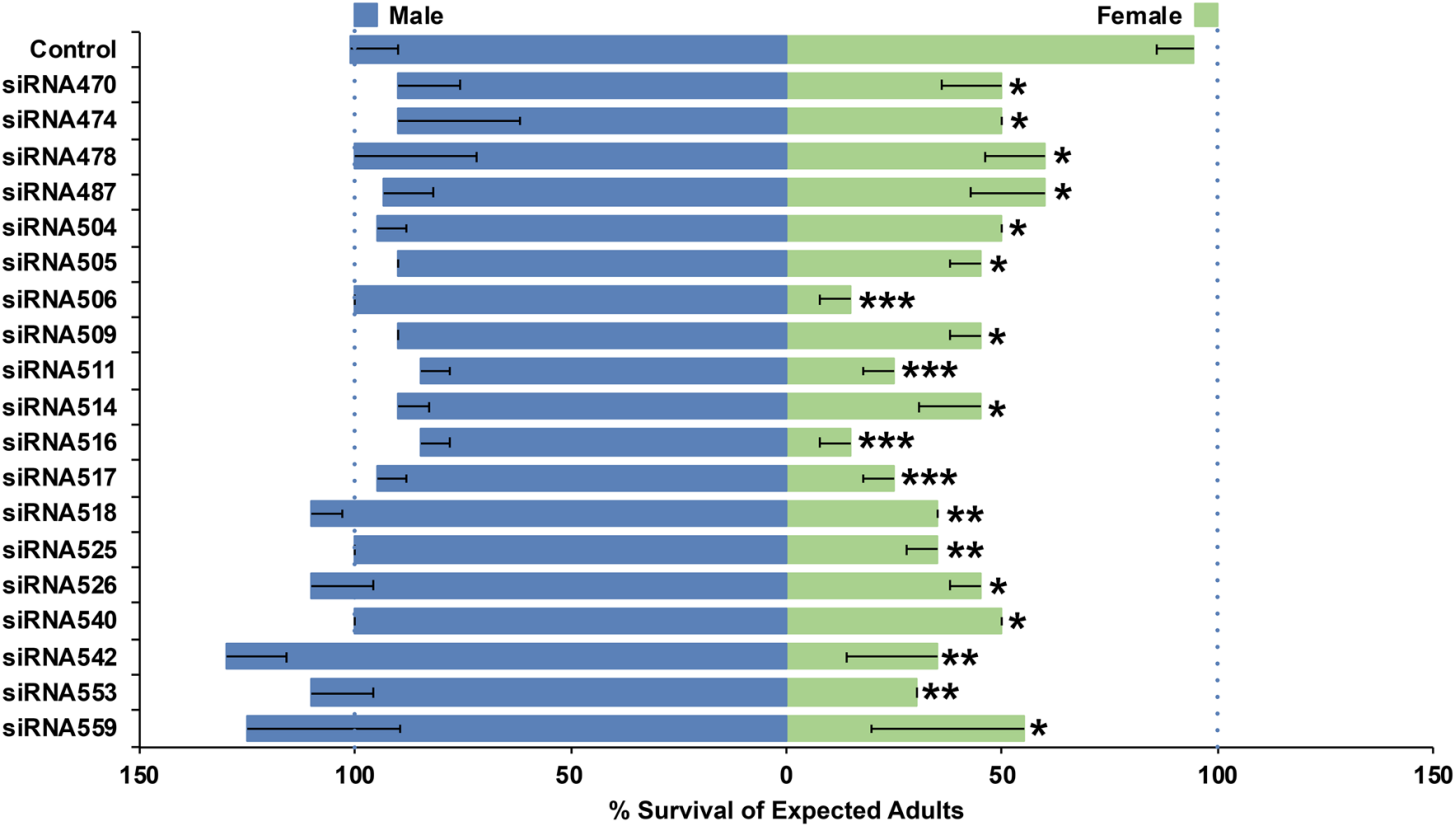
siRNAs that induce significant female-specific lethality. Significant female-specific larval mortality resulted from soaking treatments with the indicated siRNAs (* = *p* < 0.05, ** = *p* < 0.01, and *** = *p* < 0.001, Chi-square). No significant differences (*p* > 0.05, Chi-square) were observed in male survival following treatments with these siRNAs, and a control siRNA had no significant impact (*p* > 0.05, Chi-square) on survival of males or females. Data are represented as mean survival based on adult emergence, (n = 40 larvae/treatment), and error bars denote SEM.

The soaking screen uncovered a total of 19 siRNAs (Supplementary Tables S1 and S2) corresponding to M/m locus region lncRNAs that induced significant female-specific mortality and had no significant impact on male survival (Fig. 1). These siRNAs corresponded to target sites in 25 M/m locus region *lncRNA* genes, the identification numbers and chromosomal locations of which are provided in Supplementary Fig. S1 and Supplementary Table S3. Some of the siRNAs corresponded to target sites in singular M/m locus region *lncRNA* genes (Supplementary Table S1). Due to the highly repetitive nature of DNA located in this pericentric region^10^, several of the female-specific larvicidal siRNAs identified in the screen corresponded to target sites identically conserved in multiple *lncRNA* genes, at least one of which resides in the M/m locus region (Supplementary Table S2).

The *lncRNA* genes identified in the female-specific lethal screen were located throughout the M/m locus region (Supplementary Table S3). A majority (17 of 25) of the *lncRNA* genes are intergenic, though several (8 of 25) are intragenic (Supplementary Table S3). None of the *lncRNA* genes identified in the screen have known orthologs reported in Vectorbase^10^, potentially because few *lncRNA* genes have been annotated in other mosquito species. Alternatively, a recent comparative analysis of the genomes of several *Anopheles* species revealed that female-biased protein-coding genes evolve more rapidly in sequence, expression, and genic turnover than male-biased protein-coding genes; this is an atypical pattern that is proposed to have resulted from sex-specific life history challenges, such as blood feeding, that are encountered by female mosquitoes^24^, and which could also apply to *lncRNA* genes. Several of the genes were located within the M locus in a region that was not thought to be found in female (genotype m/m) mosquitoes (Supplementary Fig. S1). For example, a perfect match for the siRNA 470 target sequence is only known to reside in *AAEL026346*, which lies between the two male-specific M locus genes *myo-sex* (AaegL5_1: 151,955,864–152,241,832) and *Nix* (AaegL5_1:152,616,641–152,718,167)*.* Although siRNA 470 and the other siRNAs identified in the screen are not known to have identical target sites in mature transcripts that correspond to genes other than those noted in Supplementary Tables S1, S2, and S3, it is possible that the female-specific phenotypes observed could result, at least in part, from off-site targeting. Moreover, it is also possible that these siRNAs specifically target genes located in the known gap in the sequence at the sex determination locus, a region which has not yet been successfully sequenced but is believed to be highly repetitive^7^.

Finally, 31 of the siRNAs screened had identically conserved target sites in M/m locus region *lncRNA* genes, but had no significant impact on female or male larval survival (Supplementary Table S4). These genes (Supplementary Table S4) may lack sex-specific functions or could be active during different stages of the life cycle. It is also possible that siRNAs targeting different sites in these same genes could produce more effective silencing and yield female-specific killing. However, given the overall success of the screen, which had already identified multiple female-specific larvicides (Fig. 1), evaluation of additional siRNAs and further characterization of these particular *lncRNA* genes (Supplementary Table S4) were not pursued at this time.

### Generation of a female-specific yeast larvicide that targets M/m locus region lncRNA genes

**Figure 2:**
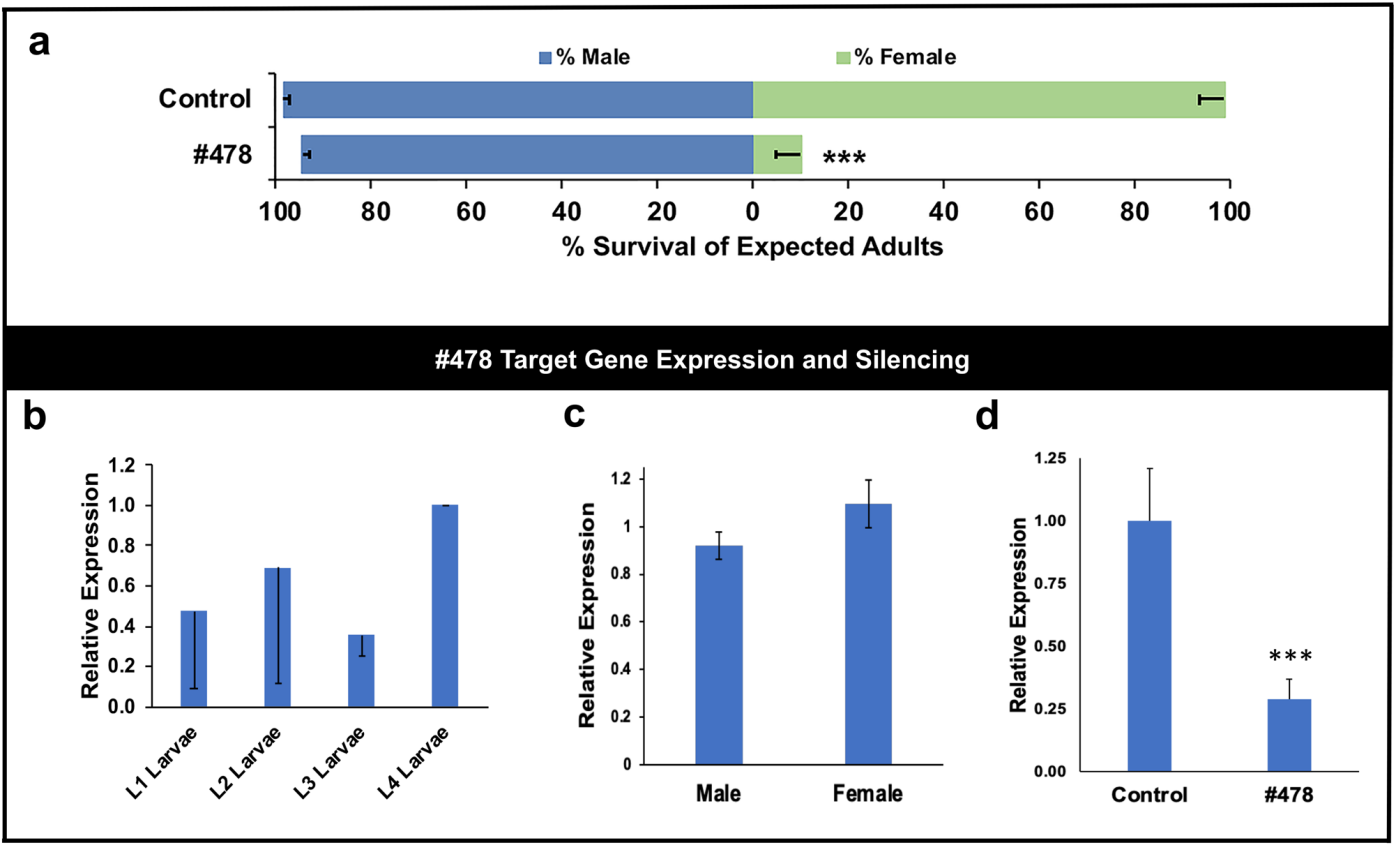
A female-specific lethal yeast interfering RNA larvicide targeting *lncRNA* genes. Significant female larval mortality resulted from oral consumption of yeast interfering RNA larvicide strain #478 [(**a**), *** = *p* < 0.001, Chi-square]. No significant death was observed in males following treatments with larvicide [(**a**); *p* > 0.05, Chi-square], and a control interfering RNA strain did not significantly impact survival of male or female larvae [(**a**) *p* > 0.05, Chi-square; data are represented as mean survival based on adult emergence following treatment of 180 total larvae, and error bars denote SEM]. (**b**–**d**) display quantification of the identical *AAEL020379, AAEL020813,* and *AAEL022952* transcripts, with mRNA levels normalized to levels of the *rpS17* housekeeping gene; error bars denote standard deviations. The #478 larvicide target transcripts are expressed throughout larval development in untreated first (L1), second (L2), third (L3), and fourth (L4) instar larvae (**b**), with no significant differences in expression levels noted between the various larval stages (*p* > 0.05, ANOVA; the expression levels are shown relative to L4). No significant differences in transcript levels were noted between third instar male and female larvae [(**c**), t-test, *p* > 0.05]. Silencing of these lncRNA targets of larvicide #478 (**d**) was confirmed through qRT-PCR (*** = *p* < 0.001 vs. target gene levels in control interfering RNA-fed larvae, Student’s t-test; error bars denote standard deviation).

In recent years, *S. cerevisiae* has been developed as a system for inexpensive and scalable manufacture of larvicidal interfering RNAs^23^. The yeast can also be used as a delivery system for RNAi larvicides to mosquitoes, in which effective gene silencing is observed in larvae that consume the larvicides in the form of inactivated yeast tablets^21–23^. Yeast RNAi larvicide technology, which could also potentially facilitate scaled culturing and sex separation of male mosquitoes, was therefore used for further characterization of several lncRNAs identified in the screen. siRNA 478, which induced significant levels of female mortality, but which did not have a significant impact on male survival (Fig. 1), was down-selected for these studies. A yeast strain designed to express a short hairpin RNA (shRNA) corresponding to the siRNA 478 target site was generated. shRNA expression was confirmed in this strain, as well as a control interfering RNA strain, through PCR amplification of cDNA corresponding to the 3’ end of each hairpin and the terminator sequence that had been prepared from total RNA extractions from each strain (Supplementary Fig. S2). Dried inactivated yeast prepared from each of these strains was fed to larvae throughout larval development. Treatment with the yeast larvicide, but not control interfering RNA yeast, resulted in significantly higher male:female ratios among the surviving mosquitoes (Fig. 2a; *p* < 0.001). Yeast larvicide #478 was therefore characterized in further detail.

When larvae were reared on yeast larvicide #478, although no significant impact on male survival was observed, only 10 ± 2% of expected adult females emerged (*p* < 0.001), with the bulk of these animals dying as fourth instar larvae (Supplementary Fig. S2b). Yeast larvicide #478 targets three M/m locus region loci: *AAEL020379, AAEL020813,* and *AAEL022952* (Supplementary Table S2). The sequences of these genes (both exons and an intron) are identical^7,10^ and correspond to a single transcript that is detected throughout larval development (Fig. 2b), is expressed at comparable levels in male and female larvae just prior to the time of death (Fig. 2c, *p* > 0.05), and which is silenced though treatment with yeast larvicide #478 (Fig. 2d; 71.1 ± 7.9% reduction in transcript levels with respect to larvae reared on control interfering RNA yeast, *p* < 0.001).

### Scaled production of adult male mosquitoes

**Figure 3:**
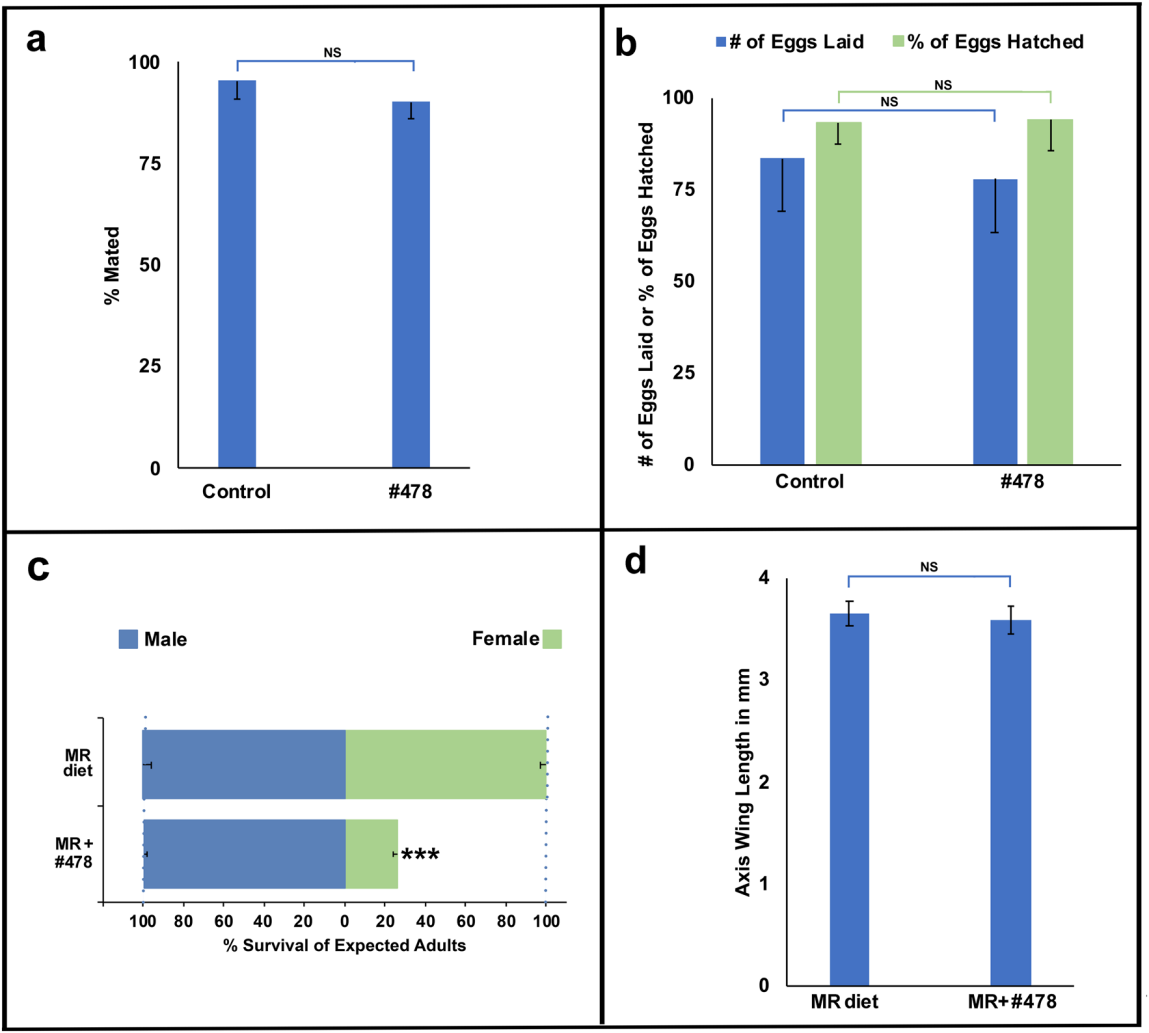
Yeast interfering RNA larvicide technology can be used for scaled production of males. Yeast larvicide #478 does not significantly impact male mating capacity [(**a**), *p* > 0.05, Student’s t-test], the number of eggs laid by females that mated with these males [(**b**), *p* > 0.05, Student’s t-test], or the percentage of larvae that hatched from these eggs [(**b**), *p* > 0.05, Student’s t-test]; results were compiled from 41 matings with #478-treated males and 72 matings with males treated with control interfering RNA yeast). Incorporation of the yeast larvicide into a larval diet used for mass-rearing (MR; n = 1200 total larvae per treatment) resulted in significant female mortality [(**c**), *** = *p* < 0.001, Chi-square] with no significant impact on male survival [(**c**), *p* > 0.05, Chi-square] or fitness [(**d**), *p* > 0.05, Student’s t-test; n = 83 control diet male wings, n = 40 #478-treated male wings]. Error bars denote SEM in all panels.

Male mosquitoes released en masse for control strategies such as the incompatible insect technique (IIT) and sterile insect technique (SIT) must successfully compete with wild-type males in areas in which they are mass-released^2,25–27^. It is therefore critical that yeast larvicides used for sex separation are specific to females and do not have undesired impacts on adult males. To examine if the impact of yeast larvicide #478 is specific to female larvae, life history traits were assessed in adult male mosquitoes that had been reared on the larvicide during larval development. Treatment with yeast larvicide #478 did not significantly impact the capacity of males to mate (Fig. 3a). The number of eggs laid (fertility) by wild-type females that mated with males treated with the larvicide, as well as the percentage of larvae that hatched from these eggs (fecundity) did not significantly differ from control male matings (Fig. 3b).

Mass rearing facilities utilize special larval diets that are optimized to produce fit male mosquitoes^28^. It is therefore helpful if yeast larvicides are compatible with these diets. Dried inactivated nutritional yeast is often a component of such diets^28^, suggesting that the nutritional yeast component could be replaced with female-specific yeast larvicides. To assess whether use of the larvicides would facilitate scaled production of males, a larval diet employed at mass-rearing mosquito facilities^28^ was modified by replacing the nutritional yeast component of the diet with dried inactivated yeast larvicide #478. The modified diet was tested on mosquitoes grown in mass-rearing trays containing 200 larvae/L of water. With respect to the control interfering RNA diet, larvicide #478 induced significant female mortality, resulting in 5 male:1 female ratios in emerging adults (Fig. 3c). The fitness of male survivors, which was ascertained through measurements of wing lengths, a proxy for body size and fitness, was not significantly different than males raised on the standard mass-rearing diet (Fig. 3d), providing further evidence that the larvicide is lethal to females, but does not impact male mosquitoes.

Although the yeast larvicides characterized here do not eliminate all females and could not be used in a stand-alone capacity, replacing nutritional yeast with the larvicidal yeast could further improve the efficacy of existing sex separation technologies^2^ or immensely reduce labor associated with hand separation strategies. Yeast interfering RNA technology, which could be implemented in remote and resource-limited locations, would likely benefit mass-rearing facilities worldwide. Moreover, the use of yeast interfering RNA larvicides would circumvent a need to further genetically manipulate existing mosquito strains that have already been developed for population control strategies, for which regulatory permits may have already been attained or might need to be acquired.

### Conclusions and potential implications for understanding the evolution of sex chromosomes in *A. aegypti*

In summary, these studies functionally verified a female larval requirement for multiple *lncRNA* genes located at the M/m locus region (Figs. 1, 2, Supplementary Fig. S1). In multiple instances, silencing *lncRNA* genes resulted in significantly increased male:female ratios that resulted from female lethality, without any significant impact on male survival or fitness (Figs. 1, 2, 3). The complete phased structure of the male M locus and the female m locus have not yet been determined, and a ~ 163 kb gap in the sequence remains^7^. Completion of the entire phased sequence will undoubtedly facilitate further interpretation and a more sophisticated understanding of these lncRNA screen data. Nevertheless, as predicted by Matthews et al*.*^7^, the availability of the existing M locus assembly has provided the opportunity to study the evolution of *A. aegypti* homomorphic sex chromosomes. These initial lncRNA studies have elucidated key findings that may help shape our understanding of sex chromosome evolution.

The evolution of sex chromosomes is believed to occur in several stages^29–31^. Initially, a homologous pair of autosomes acquires sex-determining loci, forming a proto-Y chromosome bearing a male fertility locus (M) and a dominant female suppressor (Su^F^), as well as a proto-X chromosome carrying a female fertility locus (F) and a male sterility locus (m). Suppressed recombination in the sex-determining region evolves and eventually spreads over a larger portion of the proto-sex chromosomes. The *A. aegypti* homomorphic sex chromosomes appear to have evolved into proto-sex chromosomes bearing a sex determining M/m region^3,4^ which contains a male-determining factor, *Nix*^5^, that is present on the proto-Y chromosome. Nix regulates male-specific splicing of another chromosome 1 gene, *doublesex (dsx)*, permitting expression of the male-specific splice form of *dsx*^32^ rather than the female splice form which is important for ovary development and fertility^33^. A sex-differentiated region of suppressed recombination has also evolved and is believed to have extended ~ 100 Mb beyond the M/m locus^7,34,35^.

The suppression of recombination on sex chromosomes permits accumulation of transposable elements and other non-coding sequences, as well as chromosomal rearrangements and the acquisition of sexually antagonistic genes with different alleles that differentially benefit either males or females^29–31^. Further loss of recombination between these genes and the sex-determination locus is expected to follow, eventually resulting in evolution of heteromorphic X and Y chromosomes^31^. Highly repetitive DNA, which comprises > 70% of the M locus and includes long terminal repeat retrotransposons^7^, has accumulated in *A. aegypti.* This investigation has revealed that functional *lncRNA* genes that are required in female larvae are located in this region. Given that retrotransposons can contribute to both the origin and diversification of lncRNAs^36^, one could speculate that accumulation of retrotransposons in *A. aegypti* has also contributed to the origin and diversification of M/m locus region *lncRNA* genes that evolved female-specific functions. It is predicted that these genes may eventually contribute to the formation of heteromorphic *A. aegypti* sex chromosomes and lead to genetic degeneration and reduced size of the Y chromosome^29–31^.

lncRNAs regulate a wide array of cellular activities that could contribute to sex-specific gene expression during sexually dimorphic development or differentiation, including the regulation of chromatin modifiers^12^. Although *A. aegypti* is not yet believed to possess dosage compensation, recent studies suggest that the region of non-recombination between M and m chromosomes is more extensive than previously believed^7,34,35^, suggesting that the evolution of such dosage compensation mechanisms could eventually initiate in *A. aegypti.* Interestingly, centromeric repeats in *Saccharmocyes pombe* produce dsRNA that targets formation and maintenance of heterochromatin through RNA interference (RNAi)^37^, which occurs through sequence-specific targeting of histone modifications regulated by small RNA silencing^38^. Woolcock et al.^39^ demonstrated that RNAi proteins interact with ncRNAs and retrotransposon long terminal repeats. The authors^39^ speculate that similar mechanisms could operate in other eukaryotes. Future studies will consider if lncRNAs regulate heterochromatin at the *A. aegypti* sex determination locus and elucidate the sex-specific molecular functions of lncRNAs in *A. aegypti* and other species of mosquitoes. Yeast interfering RNA technology, which may benefit efforts to mass produce male mosquitoes for emerging mosquito control programs, will likewise enhance future laboratory studies aimed at dissecting the molecular functions of mosquito lncRNAs during sex-specific development and differentiation.

## Methods

### Mosquito rearing

The *A. aegypti* Liverpool-IB12 (LVP-IB12) strain used in this investigation was reared as described^40^ in an insectary maintained at 26° C, ~ 80% relative humidity, and with a 12 h light/12 h dark cycle with 1 h crepuscular periods at the beginning and end of each cycle. Sheep blood meals (purchased from HemoStat Laboratories, Dixon, CA) were provided to adult females using a Hemotek artificial membrane feeding system (Hemotek Limited, Blackburn, UK).

### Larval soaking screen

50 custom siRNAs corresponding to target sequences in annotated *lncRNA* genes located in the M/m locus region on chromosome one (Supplementary Tables S1, S2, S3, S4) were selected using the Integrated DNA Technologies (IDT) Custom Dicer-Substrate siRNA (DsiRNA) tool^41^. In several cases, this custom siRNA design tool identified target sites that were conserved in multiple *lncRNA* target genes (Supplementary Table S2); these siRNAs were also screened to ascertain their potential use in sex-separation strategies. Custom siRNAs, as well as a control siRNA with no known target in mosquitoes^42^, were purchased from IDT (Coralville, IA). For the screen, larval soaking experiments were performed (per the methodology of Singh et al.^43^) in duplicate with 20 L1 larvae soaked in 20 ul of control or experimental siRNA at a concentration of 0.5 ug/ul for 4 h. Following soaking, larvae were reared as described^44^ in accordance with the WHO^45^ guidelines for larvicide testing. Corrections for control larval death in this assay, as well as other larvicide assays conducted in this study, were not necessary, as control mortality rates were negligible. The Chi-square test was used to test deviations from the expected survival of male and female mosquitoes (which was set at 1 male:1 female based on assessment of the sexes of untreated larvae from this strain that were reared in the insectary as described).

### Production and evaluation of yeast interfering RNA larvicides

Previously described methods for larvicidal yeast preparation^21^ were used to generate the yeast strains used in this investigation. A more detailed overview of this methodology was provided in a recent methods chapter^44^. In summary, a custom shRNA expression cassette corresponding to the siRNA #478 target sequence (Supplementary Tables S1, S2) was synthesized by Invitrogen (Carlsbad, CA) and cloned into the *pRS426 GPD* shuttle vector^46^. Following restriction digestion and sequencing to confirm the inserts, the plasmids were transformed into *S. cerevisiae CEN.PK* strain yeast (genotype *MATa/α ura3-52/ura3-52 trp1-289/trp1-289 leu2-3_112/leu2-3_112 his3 Δ1/his3 Δ1 MAL2-8C/MAL2-8C SUC2/SUC2*^47^)*,* and transformants were selected by growth on minimal media lacking uracil. shRNA expression in these strains was confirmed in each of two biological replicate trials performed as described^48^. In summary, cDNA prepared from total RNA extracted from the yeast was used as template in PCR amplifications performed with a forward primer corresponding to the 3’ end of each hairpin construct and a reverse primer corresponding to the terminator sequence in each construct. Primer sequences were as follows: Control shRNA Forward 5′-ACGCTAACATCTATCAGTGC-3′ (specific to control shRNA), #478 shRNA forward 5′-TTTATACTAATTCCAGACATTAGTC-3′ (specific to #478 shRNA), and reverse primer 5′-TCCTTCCTTTTCGGTTAGAGC-3′ (which matches the terminator sequence in all three strains). PCR products were visualized with ethidium bromide staining following gel electrophoresis. The original image of this gel (Supplementary Fig. S1) was labeled using Adobe Photoshop 2021 software.

For larvicide assays, dried inactivated yeast interfering RNA was prepared from the control and #478 strains as described^44^. As discussed in Hapairai et al.^21^, larval bioassays that conformed to the WHO^45^ guidelines for larvicide testing were performed in small container trials conducted as described^44^. In the small container assays, 20 newly hatched L1 larvae were placed in 500 ml plastic cups containing 50 ml of distilled water and a yeast tablet, which was sufficient to permit ad libitum yeast consumption throughout development. Adult emergence rates and the sexes of these adults were recorded. Control and larvicidal yeast tablets were evaluated in nine replicate container trials for each treatment. The Chi-square test was used to test for deviations from 1:1 male to female ratios, female and male survival for each treatment.

### Evaluation of male life history traits

The number of eggs produced per female (fecundity) and eggs produced per female that generated first instar larvae (fertility) were assessed as described by Hill et al.^49^ in individual females that had been mated to individual male survivors which fed on #478 or control interfering RNA yeast, or bovine liver powder (MP Biomedicals, Santa Ana, CA; also a control diet). The number of fertile females, which served as evidence of successful mating with the treated male survivors, was also recorded; for females that did not lay eggs, mating success or failure was further assessed through dissection of the spermatheca to discern the presence or absence of sperm. Results from the indicated numbers of matings combined from multiple biological replicate trials were assessed: #478-treated males (n = 41 matings), male mosquitoes fed with a standard larval laboratory diet of bovine liver powder^40^ (n = 75 matings), and males that fed on control yeast interfering RNA strain tablets (n = 72 matings). Data were analyzed through ANOVA as described^33^. Following mass-rearing trials (see below), male fitness was assessed through evaluation of wing lengths in surviving males, which were measured from the apical notch to the axillary margin, excluding the wing fringe as described^33^. Wing lengths of males combined from each of two replicate trials (see further information about experimental setup below) were compared using one-way ANOVA.

### qRT-PCR

qRT-PCR assays were performed using previously described methods^50^. In summary, total RNA was extracted using TRIzol Reagent (Invitrogen, Carlsbad, CA) from pooled or individual larvae (see further details for each specific experiment below). Total RNA was DNase treated using the DNA-free kit (Invitrogen, Thermo Fisher Scientific, Waltham, Massachusetts) according to the recommendations of the manufacturer. cDNA was prepared according to the manufacturer’s instructions using the High Capacity RNA to cDNA Kit (Applied Biosystems, Foster City, CA). Real-time quantification was performed using the Power SYBR Green PCR Master Mix (Applied Biosystems, Foster City, CA) in conjunction with an Applied Biosystems Step One Plus Real-Time PCR System. The following primer sets were used for quantification of the identical transcripts encoded by the yeast larvicide #478 target genes *AAEL020379, AAEL020813,* and *AAEL022952*: #478 forward 5′-GAAAAACGCAGT TGCGGACT-3′ and #478 reverse 5′-TGCACTTAACCTACAATGCTACA-3′. As previously described^50^, primers for the housekeeping gene *rpS17*, which was used as the internal standard for data normalization in all qRT-PCR assays performed in this investigation were: forward 5′-AGACAACTACGTGCCGGAAG-3′and reverse 5′-TTGGTGACCTGGACAACGATG-3′. All PCR reactions were performed in 3–6 replicate wells in each of two biological replicate experiments. Results from qRT-PCR were quantified by standardizing reactions to *rpS17* levels using the ΔΔCt method as described^50^.

#### Larval stage-specific analysis of lncRNA expression in untreated larvae

The analyses of lncRNA expression in different larval stages included two different pools of 10 untreated larvae of each of the following larval instars : L1, L2 L3, and L4. Total RNA from the separate pools was isolated, processed, and analyzed as described above. qRT-PCR results were standardized by setting the expression level of L4 larvae to one. Data were analyzed through ANOVA followed by Tukey’s post hoc test.

#### Analysis of lncRNA expression in untreated individual male vs. female larvae

Following preparation of total RNA from individual L3 larvae using the methodology described above, genomic DNA was isolated from each individual larva using the TRIzol reagent according to the manufacturer’s instructions. Each individual was then sexed through amplification of this genomic DNA using standard PCR assays in conjunction with the following primers, which correspond to the M locus male-specific myosin heavy chain gene *myo-sex (AAEL021838)*: forward: 5′-CGCTTTCTGGGGAAAAGGG-3′ and reverse: 5′-CTTTGGAGGCCTTGTCCTGT-3′. Following confirmation of the sex of each larva, expression of the identical *AAEL020379, AAEL020813,* and *AAEL022952* (#478 target) transcripts was performed as detailed above in individual males (n = 6) and individual females (n = 7). Data were statistically evaluated with the Student’s t-test.

#### Verification of target gene silencing

For verification of #478 yeast larvicide target gene silencing (identical transcripts *AAEL020379, AAEL020813,* and *AAEL022952*), total RNA was extracted as described above from two different pools of 20 larvae that had been reared on #478 or control yeast. qRT-PCR was then performed as detailed above using *rpS17* expression as the internal standard for data normalization. Results were expressed as fold-differences in the #478-larvicide treated larvae compared to larvae that had fed on control interfering RNA yeast. Data were statistically evaluated with the Student’s t-test.

### Mass rearing experiments

200 LVP-IB12 first instar (L1) larvae, which had been hatched from eggs placed under vacuum for 1 h to synchronize hatching, were distributed in 34 × 25 × 4 cm trays (1426B, Bioquip, Rancho Dominquez, CA) containing 1 L of distilled water. The larvae were reared on yeast + liver powder + shrimp (YSL) diet, which was developed based on the mass rearing diet of Zhang et al.^28^. This diet, which was used as a control in the present studies, consisted of 1000 mg brewer’s yeast (Spring Valley, Bentonville, AR), 250 mg of bovine liver powder (MP Biomedicals, Santa Ana, CA), and 150 mg of ground baby shrimp (Tetra GMBH, Melle, Germany) provided ad libitum. The dry components were mixed by hand into 10 ml of distilled water, producing a slurry that was stored at 4 °C. For sex-sorting experiments, the slurry was prepared without brewer’s yeast and provided to the larvae ad libitum; yeast interfering RNA tablets were fed to the larvae as follows: two tablets at L1, two tablets at L2, two tablets at L3, and four tablets at L4. The tablets were resuspended in distilled water and mixed with the liver powder-shrimp slurry, and the combined mixture was fed to larvae. Larval trays were examined daily for pupae, which were removed on the day of pupation, manually sorted by sex, and counted.

Sex ratio distortion and mortality were evaluated in three biological replicate trials, each with two replicate trays of larvae fed on YSL diets prepared with the brewer’s yeast or #478 yeast. The Chi-square test was used to test deviations from 1:1 male to female ratios for each treatment. Mortality levels for males and females for each treatment were compared according to the WHO^45^ guidelines using one-way ANOVA followed by Tukey’s post hoc multiple comparisons on data that had been arcsin transformed. Male fitness was assessed through measurement of wing lengths as described above (n = 83 control diet males; n = 40 #478-treated males).

### Ethics statement

No human subjects or vertebrate animals were used in this investigation.

## Supplementary Information

Supplementary Information.

## Data Availability

All data generated or analyzed during this study are included in this article. Yeast strains and corresponding plasmids generated in this study are available subject to completion of a material transfer agreement with Indiana University and pending procurement of any required import permits by the requesting party.
