# Supplementary material for: A functional requirement for sex-determination M/m locus region lncRNA genes in Aedes aegypti female larvae
Source: Sci Rep. 2021 May 20;11:10657. doi: 10.1038/s41598-021-90194-7 (PMC8137943; doi:10.1038/s41598-021-90194-7)
Supplement: Supplementary file 1 — Supplementary Information. [file 41598_2021_90194_MOESM1_ESM.pdf]

A functional requirement for sex-determination M/m locus region *lncRNA* genes in *Aedes aegypti* female larvae

Supplementary Files:

Keshava Mysore[1,2], Limb K. Hapairai[1,2], Ping Li[1,2], Joseph B. Roethele[1,2], Longhua Sun[1,2], Jessica Igiede[1,2], Joi K. Misenti[1,2], and Molly Duman-Scheel[1,2*]

[1]Indiana University School of Medicine, Department of Medical and Molecular Genetics, South Bend, IN, USA.

[2]University of Notre Dame Eck Institute for Global Health, Notre Dame, IN, USA.

*Contact PI:

Molly Duman-Scheel

mscheel@nd.edu

Raclin-Carmichael Hall

1234 Notre Dame Ave.

South Bend, IN 46617

(574) 631-7194

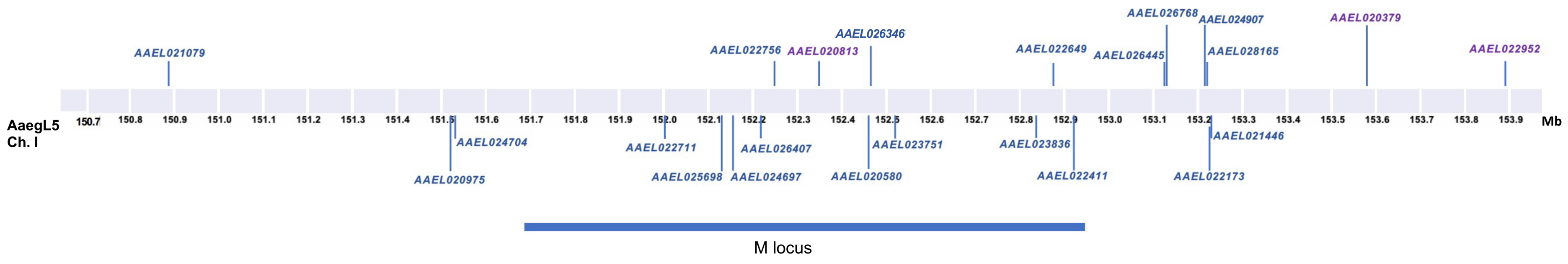

Supplementary Fig. S1. Chromosomal locations of M/m locus region genes containing target sites corresponding to the siRNAs identified in the female-specific lethal screen. Genes shown above the chromosomal schematic are on the forward strand, while those noted below it are on the reverse strand. The genes targeted by larvicide #478 are highlighted in purple. *AAEL027827* (Chromosome 1: 154,564,311-154,565,137 forward strand) and *AAEL027761* (Chromosome 1: 172,956,231-172,956,668 Mb, forward strand) also contain target sites, but are not pictured here. The predicted boundaries of the M (dark blue) locus[7] are indicated.

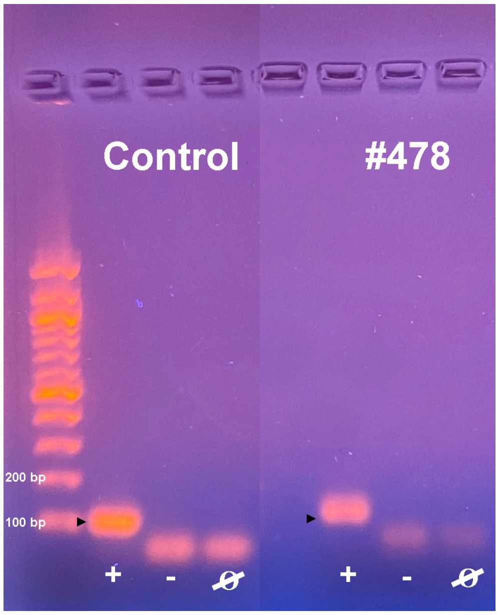

Supplementary Fig. S2. Confirmation of shRNA expression in recombinant yeast. cDNA was prepared from total RNA which was extracted from the control or #478 recombinant yeast strains. The cDNA subsequently served as template in PCR reactions in which forward primers corresponding the 3' end of the control (left) or #478 (right) shRNA hairpins and a reverse primer corresponding to the terminator amplified a fragment of ~100 bp (see DNA standard ladder at far left). Amplicons were visualized on an agarose gel stained with ethidium bromide. Negative controls included amplifications with cDNA prepared from yeast strains that had not been transformed with shRNA expression plasmids (marked by – sign), as well as a PCR reactions in which no cDNA had been added (marked by knot symbol; unused primers are visible in both of these negative controls). One of two biological replicate experiments that yielded similar results is shown here.

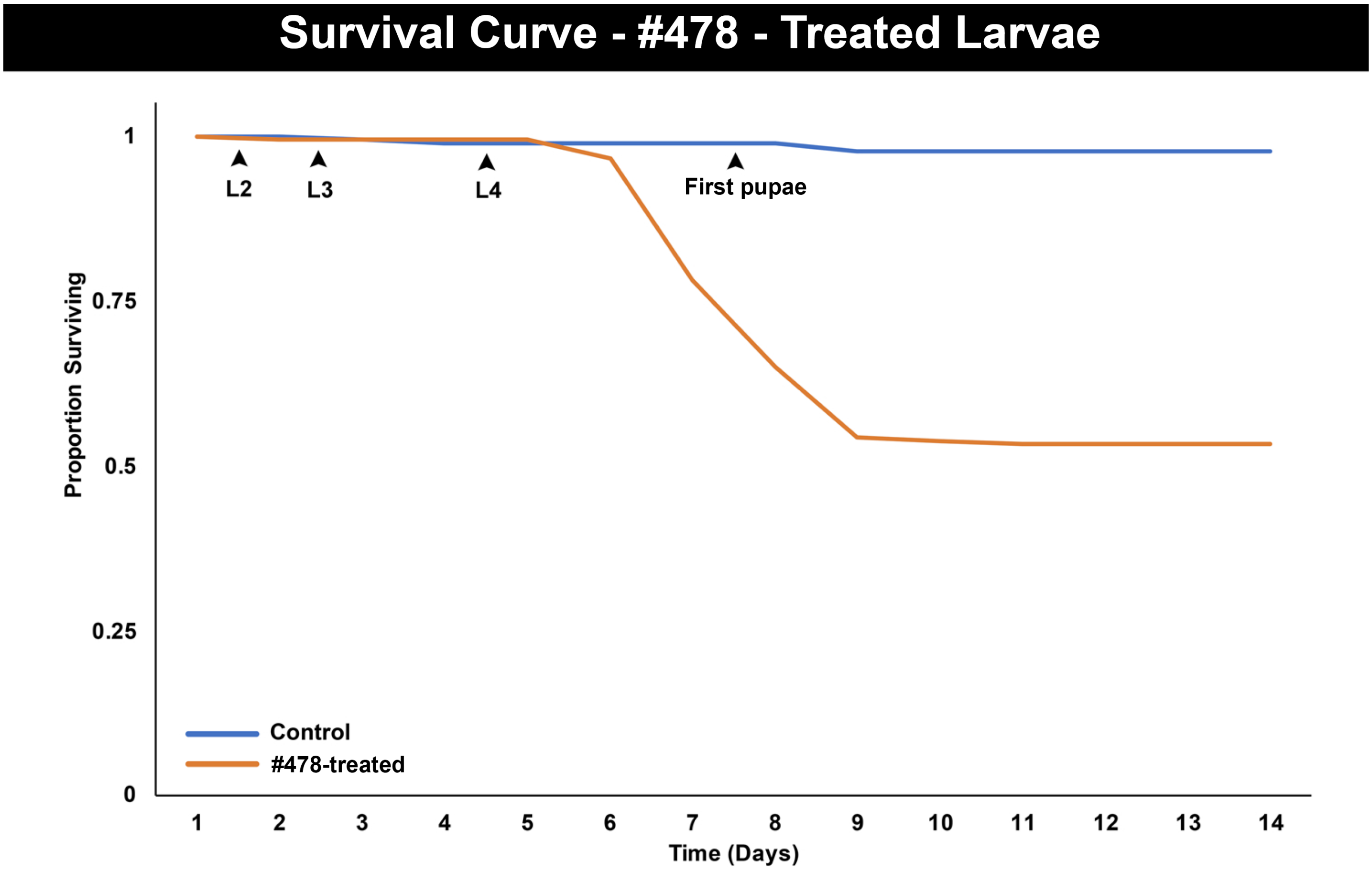

Supplementary Fig. S3. Larval survival following treatment with yeast interfering RNA larvicide #478. A survival curve for larvae that fed on yeast larvicide #478 is shown. Although larvae that were treated with control interfering RNA yeast survived, nearly half of larvae treated with yeast larvicide #478 died prior to pupation. Data were compiled from nine replicate container trials per control or #478 treatment (n = 180 larvae total per treatment). L1 = first instar, L2 = second instar, L3 = third instar, L4 = fourth instar.

siRNA #	Target Sequence	Corresponding M/m Locus Region *lncRNA* Genes
470	GGUUUACUAAAAAUCACUUUCCUUG	*AAEL026346*
487	GUCACUAAGCUCUAUAAUCAAAAUA	*AAEL022649*
511	GUUUUUAUUGUUUGCAUCAACAGUU	*AAEL023836*
525	UGGCAAAUUAUCCAAGAACAUCUAC	*AAEL028165*
526	AAAUUAUCCAAGAACAUCUACAUCU	*AAEL028165*
540	AAAGUGCAUCAUGUGAUAAAAUCGA	*AAEL021079*
542	AUUAUGAACAACAUGUUUAAAUAAA	*AAEL027827*
553	GGCUAUGCAAACCAAUUCAAAAUCA	*AAEL022711*

Supplementary Table S1. Female-specific siRNA larvicides that target single M/m locus region *lncRNA* genes. A number of siRNAs identified in the screen have perfectly conserved 25 bp target sites that are found in singular *lncRNA* genes at or tightly linked to the M/m locus.

siRNA #	Target Sequence	Corresponding M/m Locus Region *lncRNA* Genes	Corresponding *lncRNA* Genes outside M/m Locus Region
474	AGAAUCUUCUUACAAUCACUGCCUC	*AAEL020580*	*AAEL024146*
478	GACUAAUGUCUGGAAUUAGUAUAAA	*AAEL020379, AAEL020813, AAEL022952*	
504	CAUCCAACCUUCAAGCGAAUCAGTG	*AAEL026407*	*AAEL021597, AAEL022807, AAEL026655*
505	AUUGAGACUUACCAACUGAUCAGUU	*AAEL024697*	*AAEL021470, AAEL027259*
506	CAAGUGAAAAUAAACAUCAAGAUUU	*AAEL022756*	*AAEL024428, AAEL022640*
509	GAUAAAGCAUUCAUUCCGCUACUUA	*AAEL025698*	*AAEL021884*
514	AGCAGAAAGAUUGAAAUUAUUACCA	*AAEL022411, AAEL027761*	*AAEL023838*
516	AGCGUUGAAAAAUCUAUAAAAACCU	*AAEL026768, AAEL026445*	
517	AGCGAUGGAAGAUUGUAAAAAUCGA	*AAEL026768, AAEL026445*	
518	AGUCAGGGUUUAUUUCAUUGUUCGA	*AAEL021446, AAEL022173, AAEL023751, AAEL024907, AAEL028165*	*AAEL022531, AAEL027422*
559	AACCAGAAUCGGUAACCUAAAUUGU	*AAEL024704, AAEL020975*	

Supplementary Table S2. Female-specific siRNA larvicides that target multiple *A. aegypti lncRNA* genes. A number of siRNAs identified in the screen have perfectly conserved 25 bp target sites that are found in transcripts which correspond to multiple *lncRNA* genes, at least one of which resides in the M/m locus region.

Chromosomal Location	*lncRNA* Gene	Type
Chromosome 1: 150,998,186-150,999,313 forward strand	*AAEL021079*	Intragenic *(AAEL017331)*
Chromosome 1: 151,645,556-151,646,218 reverse strand	*AAEL020975*	Intragenic *(AAEL017331)*
Chromosome 1: 151,659,254-151,659,981 reverse strand	*AAEL024704*	Intragenic *(AAEL017331)*
Chromosome 1: 152,107,761-152,108,630 reverse strand	*AAEL022711*	Intragenic *(AAEL021838)*
Chromosome 1: 152,238,688-152,239,180 reverse strand	*AAEL025698*	Intragenic *(AAEL021838)*
Chromosome 1: 152,265,957-152,266,593 reverse strand	*AAEL024697*	Intergenic
Chromosome 1: 152,315,810-152,316,472 reverse strand	*AAEL026407*	Intergenic
Chromosome 1: 152,350,018-152,350,537 forward strand	*AAEL022756*	Intergenic
Chromosome 1: 152,441,886-152,442,268 forward strand	*AAEL020813*	Intergenic
Chromosome 1: 152,552,655-152,554,770 reverse strand	*AAEL020580*	Intergenic
Chromosome 1: 152,555,446-152,556,373 forward strand	*AAEL026346*	Intergenic
Chromosome 1: 152,609,464-152,609,987 reverse strand	*AAEL023751*	Intergenic
Chromosome 1: 152,908,968-152,909,549 reverse strand	*AAEL023836*	Intergenic
Chromosome 1: 152,962,655-152,995,751 forward strand	*AAEL022649*	Intergenic
Chromosome 1: 152,998,785-152,999,264 reverse strand	*AAEL022411*	Intergenic
Chromosome 1: 153,191,089-153,192,260 forward strand	*AAEL026445*	Intragenic *(AAEL014760)*
Chromosome 1: 153,195,585-153,196,259 forward strand	*AAEL026768*	Intragenic *(AAEL014760)*
Chromosome 1: 153,276,374-153,276,935 forward strand	*AAEL024907*	Intergenic
Chromosome 1: 153,284,611-153,285,229 forward strand	*AAEL028165*	Intergenic
Chromosome 1: 153,287,383-153,289,863 reverse strand	*AAEL022173*	Intergenic
Chromosome 1: 153,296,153-153,298,858 reverse strand	*AAEL021446*	Intergenic
Chromosome 1: 153,621,362-153,621,744 forward strand	*AAEL020379*	Intergenic
Chromosome 1: 153,923,572-153,923,954 forward strand	*AAEL022952*	Intergenic
Chromosome 1: 154,564,311-154,565,137 forward strand	*AAEL027827*	Intragenic *(AAEL008071)*
Chromosome 1: 172,956,231-172,956,668 forward strand	*AAEL027761*	Intergenic

Supplementary Table S3. Chromosomal locations of M/m locus region *A. aegypti* lncRNA genes identified in the screen. siRNAs that induced significant death in females correspond identically to 25 bp target sites in 24 M/m locus *lncRNA* genes, the identification numbers and chromosomal locations of which are shown. For intragenic *lncRNA* genes, the genes in which the lncRNA coding sequences are contained are noted.

siRNA #	Target sequence	M/m Locus Region *lncRNA* Genes
471	GGUUUACUAAAAAUCACUUUUCUTG	*AAEL026346, AAEL022070*
473	CACUUUUCUUGUAAUCCUCAACCAA	*AAEL022070*
475	GUGCUAUACUUGGAACUUGGCGATT	*AAEL022114, AAEL020130, AAEL024877*
476	AGAUUUCUCAGUCAACCGAAAUCGA	*AAEL022114, AAEL020130, AAEL024877*
477	CGAGGACUAAUGUCUGGAAUUAGTA	*AAEL020813, AAEL020379, AAEL022952*
479	GGAUACCGGUGGACAUUCUAUCUGG	*AAEL024877, AAEL020130, AAEL022114*
480	UGAAAGGAAUUUACGGAAGAACATT	*AAEL022112, AAEL020185*
481	GAACCAUUGUCAACAUCGAUAGUGC	*AAEL028699*
482	AACCAGUACUGAAUUGUCAGAAAGA	*AAEL022894, AAEL025485*
484	ACCAACUUAUAACAAAGAAAAGGTC	*AAEL022321, AAEL025316*
485	GUGGUGGUAAUAUAAGACGAAUCAA	*AAEL025316*
488	AGAUGAACCAUACGCUAGAACAAAG	*AAEL022649, AAEL014822*
489	GUAGUAUCAAUGAAUUAUUAUUGCT	*AAEL017331*
492	GAUGAUAUCGAACAGAAUUUUCUGA	*AAEL014760*
499	GAUGUUGAUGUACAGGUUAUUUCTA	*AAEL011832*
500	GGACCAACUUUUACUUCAGAUAAGA	*AAEL011832*
503	GGAUCAUUCUUCCCAUGCCAAUCAA	*AAEL026407*
507	CUUAGUGGAAAUGAAUUGUAGAUAC	*AAEL025579, AAEL027372*
508	AUUGUAGAUACCUGGAUGAAAUAAA	*AAEL025579, AAEL027372*
510	AAGAAUACAAAUCAUCUGCUACCAA	*AAEL021974*
512	GUAUGAGACAUGACUUAAAAAUGTT	*AAEL022650*
513	GAAGAAACUCUAUUGAUCACAUGAA	*AAEL021077*
515	GCAGCAUGAUUUCAAUACUUCACTA	*AAEL026785*
520	GAUAAAUGUUACUAAAGCUAUUCAA	*AAEL023283*
524	GGCAAAUUAUCCAAGAACAUCUACA	*AAEL028165*
527	AAACGAGAAUUUGUGGAAAUAGUTG	*AAEL026346*
528	AAACGAGAAUUUGUGGAAAUAGUTG	*AAEL026346*
541	AGCAUUUGGACAAAUCAAUACAGTA	*AAEL021079*
543	CAUGUUUAAAUAAAUAAAAAAGCAT	*AAEL027827*
544	GAGACUGCAAAGAAACGUUACUATA	*AAEL028113*
552	GAGAGAUCCAGUCCAGUUUAAUCGA	*AAEL025669*

Supplementary Table S4. siRNAs that had no significant impact on male:female ratios. A number of siRNAs corresponding to M/m locus region *lncRNA* genes were evaluated in the screen and found to have no significant impact or male or female *A. aegypti* larval survival.
